# Influence of daily oral prophylactic selenium treatment on the dibutyltin dichloride (DBTC)-induced pancreatitis in rats

**DOI:** 10.17179/excli2016-466

**Published:** 2017-02-16

**Authors:** J. Merkord, N. Görl, M. Lemke, A. Berg, H. Weber, R. Ramer, G. Hennighausen

**Affiliations:** 1 Institute of Pharmacology and Toxicology, Rostock University Medical Center, Rostock; 2 Institute of Clinical Chemistry and Pathobiochemistry, Rostock University Medical Center, Rostock

**Keywords:** DBTC, selenium, acute pancreatitis, oxidative stress

## Abstract

Dibutyltin dichloride (DBTC) is an organotin compound used as model for acute and chronic pancreatitis. Oxidative stress is one of the mechanisms of propagation of acinar cell injury in acute pancreatitis. Selenium is an essential cofactor in the antioxidant glutathione peroxidase pathway. Selenium levels are described to be subnormal in patients with acute and chronic pancreatitis. The aim of our studies was to determine the prophylactic effect of Na-selenite [5 mg kg^-1^ body weight (b.w.) per os (p.o.) 7 days] on the pathogenesis and course of DBTC- induced pancreatitis. Male inbred rats (LEW-1W Charles River) of 150 g body weight were used in this study. Experimental pancreatitis was induced by intravenous administration of 6 mg kg^-1^ b.w. DBTC in rats. Na-selenite was administered as daily oral dose of 5 mg kg^-1^ b.w. 7 days before induction of DBTC-pancreatitis. Malondialdehyde (MDA) was measured for monitoring levels of oxidative stress. Elimination of DBTC was reflected as tin concentration in bile and urine. Organ changes were indicated by serum parameters as well as histology. A prophylactic Na-selenite application significantly diminished MDA- and bilirubin concentration in serum, activities of lipase and transaminases as well as organ injuries compared to DBTC- treated rats in the absence of Na-selenite. The prophylactic oral treatment with Na-selenite in the scope of DBTC-induced pancreatitis points to a reduced oxidative stress characterized by diminished MDA serum levels and a milder course of pancreatitis suggesting prophylactic substitution with Na-selenite to probably elicit beneficial effect on the clinical outcome in patients with endoscopic retrograde cholangiopancreatography (ERCP).

## Introduction

Dibutyltin dichloride (DBTC) is an organotin compound serving as model system for analyses of toxicological investigations due to various pathological effects on the organism. In recent years the industrial use of organotin and its metabolites as stabilizers in polyvinylchloride manufacturing, as biocides in agriculture and in antifouling paints has become strongly restricted by laws of the European Union (Kimmel et al., 1977[11]; Merkord, 1994[18]; Gies et al., 2000[8]). 

Several studies focussed on the acute and chronic toxic effects of DBTC at doses of 6 mg kg^-1^ or 8 mg kg^-1^ b.w. i.v. Depending on time and dose an acute interstitial pancreatitis, cholestasis with bile duct and liver lesions, depression of the thymus-dependent immune system accompanied with thymus atrophy were observed in rats (Barnes and Magee, 1958[2]; Hennighausen et al., 1980[10]; Merkord et al., 1982[20]; Merkord and Hennighausen, 1989[21]; Merkord et al., 1991[21], 1995[23]). The mechanisms of DBTC-induced pancreatitis were discussed in a former publication from our group (Merkord et al., 1997[22]). Biochemical effects of organotin compounds like DBTC are interactions at dithiol groups, inhibition of mitochondrial oxidative phosphorylation and glutathion-S-transferase activity, as well as activation of caspase activity and induction of apoptosis at low concentrations (Pennings et al., 1983[28]; Gennari et al., 2002[7]). The major parameter conferring DBTC-induced pancreatitis is a high concentration of organotin in bile after administration of DBTC. 4 hours after application, elevated levels of organotin were measured in different compartments, especially in bile, liver and pancreas (Merkord et al., 1997[22], 2000[25]). The excretion rate of DBTC in urine is low, but appears higher in combination with chelating agents after 1 to 4 days (Merkord et al., 2001[26]).

Selenium is an essential trace element, which can be found in different concentrations in soil and food, especially in yolk of an egg, in fish, meat and innards (Kimmel et al., 1977[11]). Its deficiency is associated with skeletal diseases and cardiac myopathy and may increase the risk for cardiovascular diseases and for cancer (Salonen et al., 1985[32]; Neve et al., 1985[27]; Albrecht et al., 1994[1]).

Selenium is a modulator of prostaglandin-depended reactions and has antidotal effects against nuclear radiation (Schrauzer, 1990[35], 1998[34]). Furthermore selenium, as an essential component of glutathione peroxidase, plays a critical role in protecting the organism against cell injury caused by radical oxygen species (Neve et al., 1985[27]; Saint-Georges et al., 1989[31]; Zimmermann et al., 2000[50]). Furthermore selenium has been postulated to exert anticarcinogenic and antimutagenic properties (Schrauzer, 1990[35], 1998[34]).

Oxidative stress is one of the mechanisms of propagation of acinar cell injury in acute pancreatitis (Birk et al., 1995[3]; Schoenberg et al., 1992[33]; Schulz et al., 1999[36]). Decreases of serum selenium levels as well as activity of glutathione peroxidase were found to be associated with acute and chronic pancreatitis (Kuklinski et al., 1991[13], 1995[14]; Wollschläger et al., 1999[47]; Gärtner et al., 1997[6]; McCloy, 1998[17]; Scolapio et al., 2004[37]; Zelck and Karnstedt, 1993[49])

In experiments using DBTC-induced pancreatitis in rats, low therapeutical doses of selenium failed to increase the reduced glutathione peroxidase activity. On the other hand, high doses of selenium led, independent of state of health of rats, to an increase of glutathione peroxidase in serum (Frommhold et al., 1997[5]). Selenium has not yet been found to target other antioxidative enzymes such as superoxide dismutase (Wollschläger et al., 1997[46]; Zelck and Karnstedt, 1993[49]).

Thus taken together the anti-oxidative treatment of acute pancreatitis is still controversially discussed (Birk et al., 1995[3]; Kuklinski et al., 1995[14]; Wollschläger et al., 1999[47]; Gärtner et al., 1997[6]; Sevillano et al., 2003[39][38]; Virlos et al., 2003[44]; Stopinski et al., 1994[43]).

Selenium has been reported as a crucial modulator of inflammatory reactions. On the one hand it activates the transcription factor AP-1, which induces a controlled inflammation and on the other hand it inhibits NF-κB, and prevents a massive inflammation (Zimmermann et al., 2000[50]; McCloy, 1998[17]; Forceville et al., 1998[4]; Zimmermann et al., 1997[51]). Another way of diminishing the toxic reactions of metals and organometals in the organism could be the formation of complexes consisting of selenium and noxious substances. As such selenium decreases the toxicity of mercury in vivo via formation of albumin-mercury-selenite complexes accompanied by increasing mercury elimination in urine (Schrauzer, 1990[35], 1998[34]; Wu et al., 1990[48]; Hennighausen and Lange, 1980[9]; Potter and Matrone, 1977[29]). Accordingly, selenium complexes with DBTC as cause of the reduced toxicity appear feasible. An alternative explanation for the reduction of DBTC-induced toxicity may lie in a probable reduction of DBTC-induced lipid peroxidation by selenium. Lipid peroxidations are part of reactions that induce oxidative stress which is characterized by the concentration of malondialdehyde in serum. 

The purpose of this study was to evaluate the effect of prophylactic applications of Na- selenite as a possible antioxidant preventive therapy in acute DBTC-induced pancreatitis and the protective effect of Na-selenite in diminution the toxicity of DBTC in rats. There is the possibility that the administration of Na-selenite increases the selenium levels in serum concomitant with higher activities of glutathione peroxidase. Towards these points the oxidative stress in scope of DBTC-induced pancreatitis could be lowered by a prophylactic treatment by selenium. This fact is interesting on the clinical outcome in patients with ERCP.

We investigated the influence of a daily oral prophylactic application of Na-selenite over 7 days on the acute toxic effects of a single dose of DBTC of 6 mg kg^-1^ DBTC i.v. in rats.

The pathohistological changes of bile duct, liver, pancreas and thymus were examinated by light microscopy 2, 4, 7, 14 and 28 days after treatment.

Moreover, pathobiochemical parameters of pancreatitis (amylase and lipase activity), oxidative stress (malondialdehyde in serum), liver lesions (bilirubin and transaminases) and cholestasis (alkaline phosphatase) were determinated.

## Methods

### Animals and reagents

8 weeks old male inbred rats (LEW-1W, Charles River) of 150 ± 20 g body weight were randomly allocated to one of 3 groups (n = 6 / group). The experimental groups were as follows:

- Group 1 (Na-selenite): Na-selenite 5 mg kg^-1^ b.w. p.o. daily in a period of 7 days;
- Group 2 (DBTC): Single dose of 6 mg kg^-1^ b.w. DBTC i.v.;
- Group 3 (Na-selenite + DBTC): Na-selenite (5 mg kg^-1^ b.w. p.o. daily in a period of 7 days before DBTC application in a single dose of 6 mg kg^-1^ b.w. DBTC i.v.). The experiments cover a period of 4 weeks after DBTC-administration for all three test groups.

Na-selenite was dissolved in sodium chloride for oral administration. For intravenous administration DBTC (Sigma-Aldrich Chemie GmbH, Steinheim) was first dissolved in 100 % ethanol. Two parts of this solution were mixed with three parts glycerol. The animals were sacrificed 2, 4, 7, 14 and 28 days after the day of DBTC application.

Blood collected from the retroorbital plexus of rats was examined before and 7 days after Na- selenite administration and 1, 2, 4, 6 and 24 hours after DBTC administration for MDA concentration in serum.

For measurement of biliary excretion of tin, animals (five per group) were anaesthetized with pentobarbital (50 mg kg^-1^ b.w. i.p.) and a midline laparotomy was performed. The bile duct at the hilium of the liver was cannulated with PE 10 polyethylene tube for a distance of 0.5 cm. Afterwards, one group was treated with a single dose of 6 mg kg^-1^ b.w. DBTC i.v. and the other group with Na-selenite (5 mg kg^-1^ b.w. p.o. 1 hour before DBTC [6 mg kg^-1^ b.w. i.v.] application). The bile was collected after DBTC and Na-selenite + DBTC administration every 60 min of an experimental period of 4 hours.

### Analyses of morphology

Tissue samples of biliopancreatic duct, pancreas and liver were fixed in calcium formalin and processed for histopathological examination. Paraffin sections were stained with haematoxylin and eosin.

### Analytical methods

Blood samples for the determination of malondialdehyde (MDA) were drawn after 1, 2, 4, 6 and 24 hours, for the determination of serum alkaline phosphatase, bilirubin and transaminases were drawn after 1, 2, 4, 7, 14 and 28 days and for amylase and lipase 1, 2, 4 and 7 days after administration of DBTC. 7 days before and at the day of DBTC application we measured these enzymes in serum to identify the best time point to start the measurements.

The amylase and lipase activities in serum of rats were measured using a commercially available test kits from Roche Diagnostics GmbH (Mannheim, Germany).

Bilirubin was determined by the DPD (2.5-dichlorphenyl diazonium salt) method (37° C).

Alkaline phosphatase was measured by an optimized method of the German Society of Clinical Chemistry (37° C) with p-nitrophenylphosphat as substrate. All these parameters were measured on the analyzer Hitachi 717 with test kits from Roche Diagnostics. Transaminases were measured with test kits from Sigma-Aldrich (Steinheim, Germany). The MDA concentration was measured by HPLC with a test kit from Chromosystems GmbH (Munich, Germany).

The serum levels of selenium and the concentrations of tin in bile, urine, pancreas, liver and thymus were estimated by atomic absorption spectrometry (AAS) after microwave-induced digestion (Merkord et al., 2000[25]). For the electrothermal determination of tin at 286.5 nm, an AAS GBC 908 AA was used as previously described (Merkord et al., 1997[22]).

### Statistical analysis and evaluation

Means of normally distributed data with similar variances were compared by Student's t-test. In the case of significant differences in variance, means were compared by Welch's t test. Non-normally distributed data were evaluated by Wilcoxon test. p < 0.05 was considered as significant (Sachs, 1992[30]).

## Results

### Morphological modulation of the biliopancreatic duct by Selenium and DBTC

**Figure 1:**
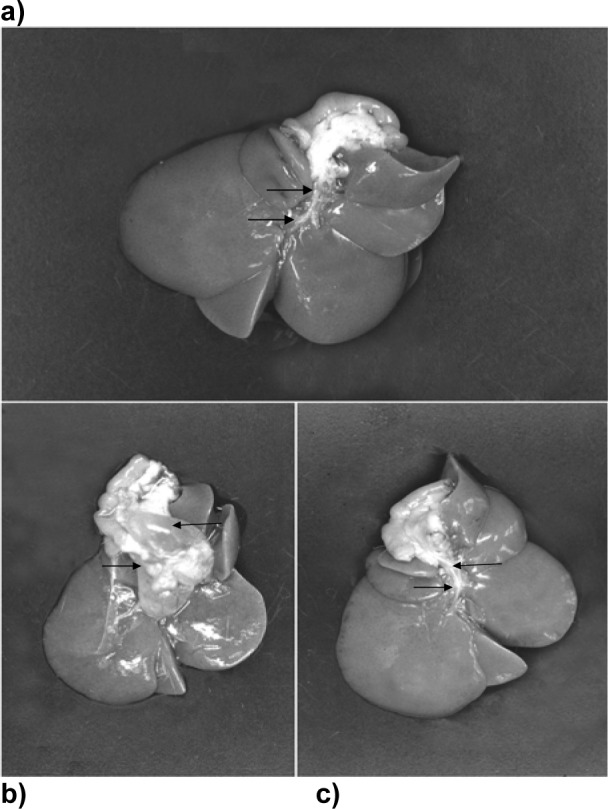
Impact of Na-selenite on DBTC-induced dilatation of the biliopancreatic duct. a) Biliopancreatic duct 7 days after Na-selenite administration. b) Biliopancreatic duct 7 days after DBTC administration. c) Biliopancreatic duct 7 days after Na-selenite + DBTC administration. Arrows indicate the biliopancreatic duct in the respective image.

In a first attempt to observe morphological changes induced upon treatment with DBTC, visible changes of the biliopancreatic duct were investigated. As expected for the DBTC- treated group, the biliopancreatic duct was extremely dilated. Total necrosis of the surface epithelium of the duct let to adhesion and obstruction of the duct followed by cholestasis (Figure 1b(Fig. 1)) whereas in the Na-selenite-treated group, the biliopancreatic duct was not dilated (Figure 1a(Fig. 1)). The prophylactic administration of Na-selenite diminished the DBTC-induced lesions of the biliopancreatic duct (Figure 1c(Fig. 1)).

### Inhibition of the DBTC-induced pathological modulation of pancreas and liver by selenium

To further analysize morphological changes induced upon DBTC treatment and prophylactic Na-selenite administration, section from pancreas and liver were stained with hematoxilin/eosin to visualize the toxic impact of the test substances.

Two days after DBTC treatment acute interstitial pancreatitis with inflammatory cells, dilated acinar lumina and scattered acinar cell necrosis had developed. In the liver, edemas of the portal tracts were the only signs of damage while in the combined treatment group of Na-selenite and DBTC the lesions appeared milder than in the DBTC-group (data not shown).

**Figure 2:**
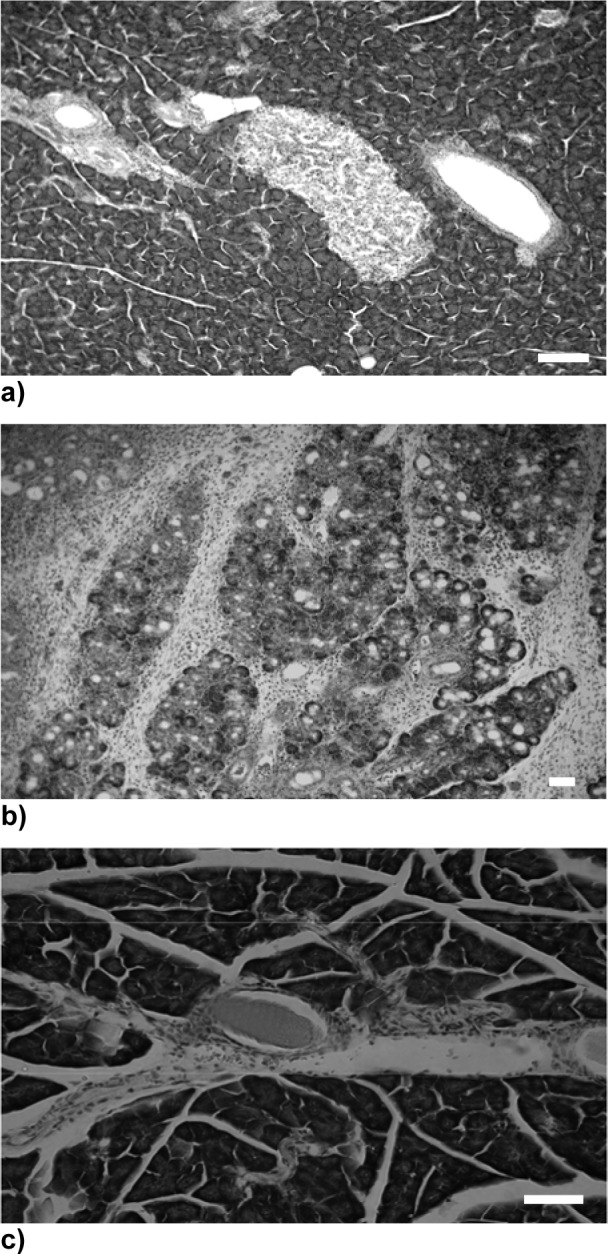
Influence of Na-selenite on DBTC-induced acute interstitial pancreatitis. Images depict hematoxilin/eosin staining of a normal structure of pancreas in Na-selenite-treated rats (a, magnification x 80) whereas DBTC-induced acute interstitial pancreatitis (b, magnification x 40). In rats treated with combination of DBTC and Na-selenite a mild interstitial pancreatitis can be noticed (c, magnification x 120). Pictures were taken 7 days after administration of the indicated test substances, respectively. Scale bars indicate 100 µm, respectively.

Following the treatment period of 7 days none of the Na-selenite treated rats exhibited morphological abnormalties in the pancreas (Figure 2a(Fig. 2)). However, DBTC treatment caused an acute interstitial pancreatitis with infiltrates of eosinophilic granulocytes, histiocytes, lymphocytes, macrophages and plasma cells (Figure 2b(Fig. 2)) that was reversed when rat were pretreated with Na-selenite (Figure 2c(Fig. 2)).

**Figure 3:**
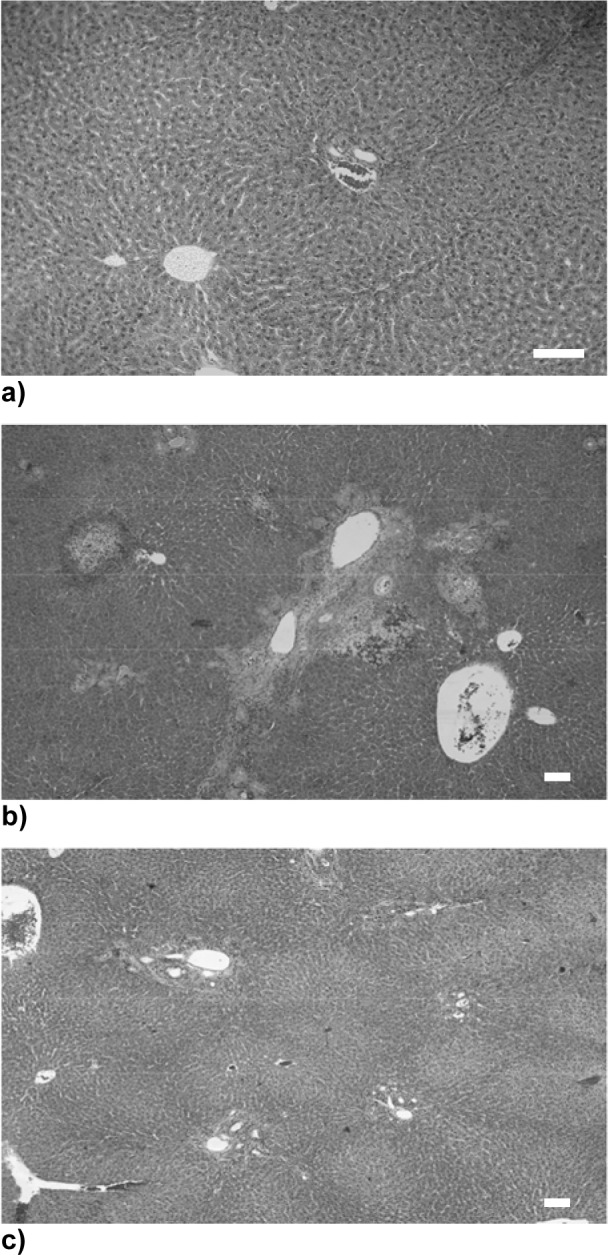
Influence of Na-selenite on DBTC-induced liver malfunction. Images depict hematoxilin/eosin stainings of a normal structure of the liver in Na-selenite-treated rats (a, magnification x 80) whereas DBTC caused inflammation, isolate necrosis and intrahepatic bile duct hyperplasia (b, magnification x 40). In rats treated with combination of DBTC and Na-selenite the DBTC- induced inflammation, necroses and intrahepatic bile duct hyperplasia were reduced (c, magnification 40). Pictures were taken 7 days after administration of the indicated test substances, respectively. Scale bars indicate 100 µm, respectively.

Similar developments were observed in the liver. Accordingly, Na-selenite treated rats showed no pathological morphology in liver tissue (Figure 3a(Fig. 3)). DBTC induced an acute inflammation of portal tracts and parenchyma necrosis in the perivenular zone in the liver (Figure 3b(Fig. 3)). As expected from the effects of Na-selenite pretreatment on pancreatic pathogenensis induced by DBTC, no signs of morphological changes in liver were observed (Figure 3c(Fig. 3)).

After 28 days one third of the DBTC-treated animals developed periductal and interstitial fibrosis in the pancreas and obstruction until the end of the biliopancreatic duct. The blockade was maintained by necrotic material of the biliopancreatic duct wall, increased by condensed secretion and cell infiltration. In the liver bile duct hyperplasia and necrosis were present. 28 days after combined treatment of Na-selenite and DBTC, no signs of morphological changes in pancreas and liver were observed (data not shown).

### Distribution of tin and selenium in rats

To further characterize the distribution pattern DBTC in the bodies of rats, concentration of tin was determined in bile, liver and pancreas. These evaluation further provide data concerning the elimination of DBTC alone or in presence of Na-selenite. In the DBTC-group, the highest tin concentrations were measured in the collected bile and in the organs (liver and pancreas) 4 hours after administration of DBTC (Table 1(Tab. 1)). 7 days after DBTC application the highest tin concentrations were detected in livers. This corresponds with the toxic effect of DBTC in this organ. Na-selenite administration diminished the tin concentration in bile, pancreas and liver in DBTC-treated rats 4 hours after DBTC administration. 7 days after DBTC-administration, Na-selenite increases the tin concentration in urine. After this period Na-selenite had no effects on pharmacokinetic parameters of tin, regarding to distribution and elimination. In the Na-selenite-group no higher tin levels were observed.

Selenium concentrations were found after 4 hours almost equal in pancreas and liver as well as in urine and bile regardless of treatment groups. However, after 7 days we found selenium accumulation in the Na-selenite-group in pancreas and liver (Table 2(Tab. 2)). In the Na-selenite + DBTC-group the concentrations in pancreas and liver were lower, probably indicating tin- selenite complexes, which increases the tin elimination and reduced the toxicity in the organs (Table 2(Tab. 2)).

Thus prophylactic administration of Na-selenite diminished the DBTC-induced lesions of the biliopancreatic duct, pancreas and liver associated with Na-selenite-enhanced elimination of tin.

Further experiments were carried out in order to monitor biochemical parameters to quantify pancreatitis, liver damage, cholestasis and oxidative stress.

### Inhibition of the DBTC-induced oxidative stress by Na-selenite

**Figure 4:**
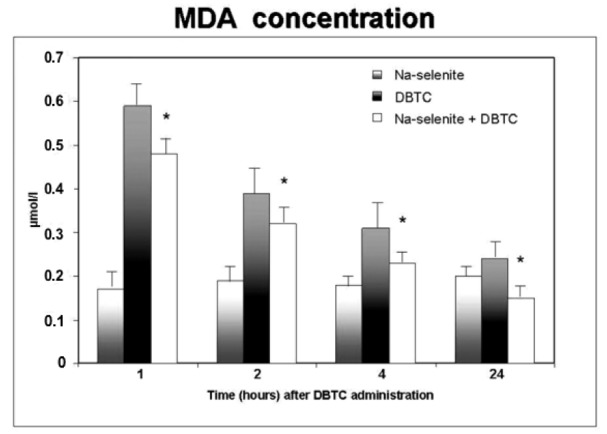
Modulation of serum MDA concentrations by DBTC and Na-selenite. MDA-concentration were measured in serum of rats 1, 2, 4 and 24 hours after administration of Na-selenite, DBTC and Na-selenite + DBTC. Data indicate mean values ± SD of n = 6 rats in each group, *P < 0.05 for comparison of DBTC vs. corresponding Na-selenite + DBTC group.

In a first attempt to monitor the modulation of the DBTC-induced toxicity by Na-selenite, serum malondialdehyd as marker of oxidative stress was evaluated. The DBTC-treated rates revealed increased serum concentrations of MDA after 1, 2 and 4 hours with highest levels after 1 hours that constantly decreased within the 24 hours time frame (Figure 4(Fig. 4)). Na-selenite significantly inhibited the DBTC-induced MDA concentrations in serum at all time points measured. The MDA levels in the Na-selenite-group remained virtually unaltered.

### Inhibition of DBTC-induced acute pancreatitis by Na-selenite

**Figure 5:**
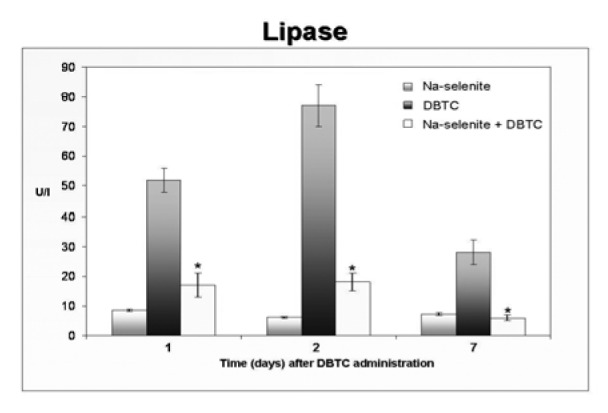
Modulation of serum lipase by DBTC and Na-selenite. Activity of lipase was quantified in serum of rats 1, 2 and 7 days after administration of Na-selenite, DBTC and Na-selenite + DBTC. Mean values ± SD, n = 6 in each group. Data indicate mean values ± SD of n = 6 rats in each group, *P < 0.05 for comparison of DBTC vs. corresponding Na-selenite + DBTC group.

As a next step, the lipase activity was measured as pathobiochemical parameter of pancreatitis. In agreement with our observed morphological changes indicating acute pancreatitis induced upon DBTC treatments, serum levels of lipase appeared strongly elevated in the DBTC-group following 1, 2 and 7 days (Figure 5(Fig. 5)). Lipase in serum of the DBTC + Na-selenite group was significantly lower compared to the DBTC-group. In serum from rats treated with Na-selenite alone minimal lipase was detected, respectively. 

### Inhibition of DBTC-induced cholestasis by Na-selenite

**Figure 6:**
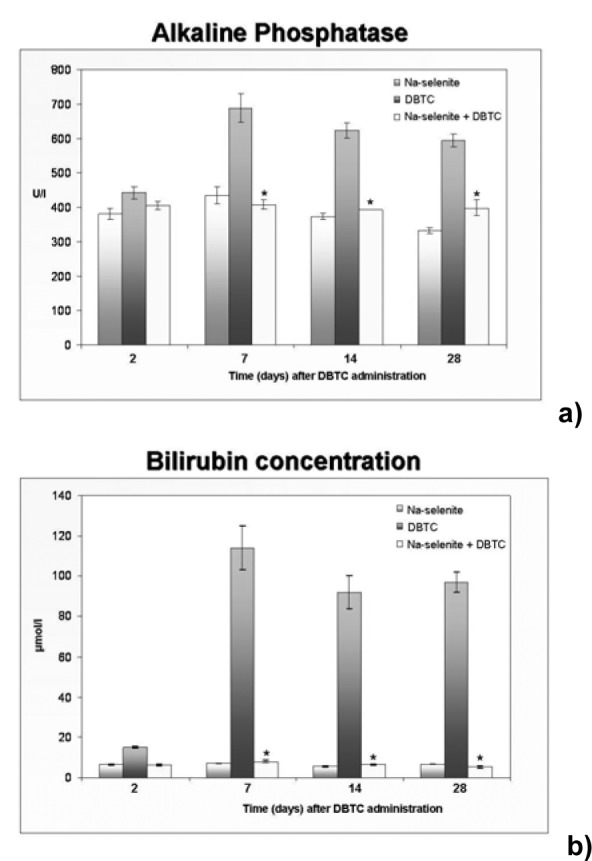
Modulation of serum alkaline phosphatase and bilirubin by DBTC and Na-selenite. Alkaline phosphatase activity (a) and bilirubin concentrations (b) were determined in serum of rats 2, 7, 14 and 28 days after administration of Na-selenite, DBTC and Na-selenite + DBTC. Data indicate mean values ± SD of n = 6 rats in each group, *P < 0.05 for comparison of DBTC vs. corresponding Na-selenite + DBTC group.

The increase in alkaline phosphatase activity (AP) and bilirubin concentration in serum between 2 and 28 days after treatment with DBTC clearly indicates induction of a cholestasis (Figure 6(Fig. 6)). 

Na-selenite significantly reduced the AP activity (Figure 6a(Fig. 6)) and the concentration of bilirubin (Figure 6b(Fig. 6)) in serum 7 to 28 days after DBTC administration in comparison to the DBTC-group. The rats in the Na-selenite-group showed no pathological levels of bilirubin and AP and their levels were comparable to the Na-selenite + DBTC-group.

### Reduction of DBTC-induced liver damage by Na-selenite

**Figure 7:**
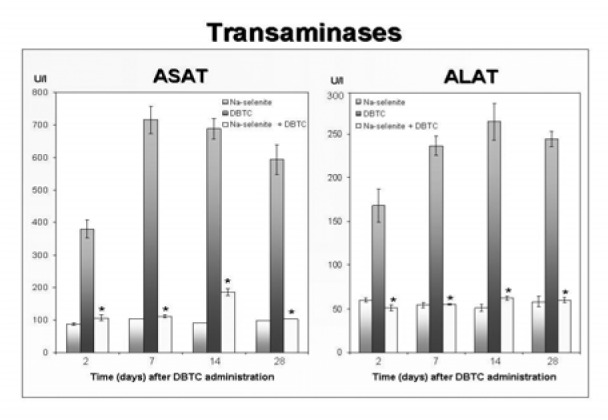
Modulation of serum transaminases by DBTC and Na-selenite. Transaminases (Alanin- Aminotransferase [ALAT] and Aspartat-Aminotransferase [ASAT]) were quantified in serum of rats 2, 7, 14 and 28 days after administration of Na-selenite, DBTC and Na-selenite + DBTC. Data indicate mean values ± SD of n = 6 rats in each group, *P < 0.05 for comparison of DBTC vs. corresponding Na-selenite + DBTC group.

DBTC significantly increased both transaminases (Alanin-Aminotransferase [ALAT] and Aspartat-Aminotransferase [ASAT]) that were monitored in a time frame between 2 and 28 days after administration. Again, in the combination group (Na-selenite + DBTC) transaminases were significantly decreased as compared to the DBTC-treated rats at all time points monitored in (Figure 7(Fig. 7)).

## Discussion

In former studies we described the diminution by combined intravenous and oral selenium treatment on bile duct-, pancreas-, liver- and thymus lesions in rats after DBTC application (Lemke et al., 2006[15]). These results favoured the decreasing effects of selenium on oxidative stress, monitored by MDA levels, as an important mechanism in scope of DBTC-induced pancreatitis.

The significant decrease of MDA in serum 2, 4, and 24 hours after DBTC administration caused by selenium suggests a mechanism that confers reduction of oxidative stress in acute DBTC-induced pancreatitis. Oxygen radicals have been implemented in the pathogenesis of acute pancreatitis (Schoenberg et al., 1992[33]; Schulz et al., 1999[36]). In previous studies we found an imbalance between oxidants and antioxidants in pancreas in the scope of DBTC-induced pancreatitis (Weber et al., 1995[45]). The early morphological changes observed in DBTC-pancreatitis may be a consequence of lipid peroxidation.

The prophylactic treatment with Na-selenite significantly diminished the MDA concentration in serum following 2, 4 and 24 hours after DBTC application and had an effect on the lipid peroxidation. In comparison to our former study, where Na-selenite was administered after induction of DBTC-pancreatitis, prophylactic administrations of Na-selenite elicited a faster and more efficient impact on oxidative stress, characterized by significantly decreased MDA- levels in serum. Application of Na-selenite in the absence of DBTC had no pathomorphological effects on bile duct, pancreas, liver and thymus and did not increase serum parameters like amylase, lipase, bilirubin, transaminases and MDA. Lower selenium levels were postulated to occur in inflammatory reactions (Kuklinski et al., 1991[13], 1995[14]; Kraft et al., 1995[12]; Wollschläger et al., 1999[47]; Gärtner et al., 1997[6]; McCloy, 1998[17]; Scolapio et al., 2004[37]). Therefore the activity of the selenium-dependent glutathione peroxidase, an important enzyme of endogenous antioxidant defence, is diminished (Neve et al., 1985[27]; Saint-Georges et al., 1989[31]; Zimmermann et al., 2000[50]). Prophylactic application of Na-selenite prior to an inflammatory reaction such as pancreatitis may increase the activity of the selenium-dependent glutathione-peroxidase in serum. This hypothesis is supported by the results of our measurements of selenium in serum before and after application of Na-selenite and induction of a DBTC-pancreatitis. Increase of selenium levels following application of Na- selenite may lead to a higher expression of glutathione-peroxidase and thus to an enhanced enzymatic antioxidant defence in scope of DBTC-induced pancreatitis. The enhanced enzymatic antioxidant defence could be one of the key factors responsible for the minimal pancreas and liver damages after selenium treatment.

A further explanation for the observed lower toxicity of DBTC after selenium administration could be the decrease of organotin compounds in the bile caused by enhanced generation of water soluble adducts with selenium and a higher and delayed excretion by the kidney (Table 2(Tab. 2)), as has been reported before (Lemke et al., 2006[15]). Moreover, it cannot be excluded that selenium forms unknown complexes with DBTC, which are not measurable by atomic absorption spectrometry.

In vivo experiments revealed selenium to react with mercury and form albumin-mercury- selenite complexes, thereby conferring enhanced mercury elimination, reduction of pathohistological changes in organs and mercury-induced lethality (Schrauzer, 1990[35], 1998[34]; Wu et al., 1990[48]). A similar mechanism of action appears feasible in terms of the presented decrease of DBTC-induced toxicity by selenium. Accordingly, we did not find higher tin concentrations in urine after Na-selenite + DBTC administration after 4 hours and 4 days but after 7 days in comparison with the DBTC-group (Table 1(Tab. 1)) suggesting a delayed enhancement of organotin elimination by selenium.

After DBTC administration, high concentrations of organotin (10^-5^-10^-6^ M) are excreted into the bile and induce cytotoxic effects on the biliopancreatic duct epithelium (Merkord et al., 1982[20]). Partial necrosis of the surface epithelium of the biliopancreatic duct after administration of DBTC leads to the formation of plugs and concrements.

The consequence of these biliopancreatic duct injuries is obstruction of the duct, cholestasis, pancreatic reflux, pancreatitis and liver lesions (Merkord et al., 2003[24]).

In our studies the biliary excretion of tin was reduced 4 hours after DBTC treatment in the Na-selenite + DBTC-group compared to the DBTC-group (Table 1(Tab. 1)). The urinary excretion was increased 7 days after DBTC treatment in the Na-selenite + DBTC-group compared to the DBTC-group (Table 1(Tab. 1)). Thus the formation of tin-selenite complexes appears likely.

In other studies, we analyzed the local cytokine profile and infiltrating lymphocytes in this DBTC-rat model of pancreatitis. IL-1ß, IL-6, IL-5 and IL-10 were immediately up regulated in the acute phase of disease, while lymphocyte-restricted expression of IL-2, IL-2R and IFN-y was only found in the chronic course (Sparmann et al., 2001[40]). Studies concerning the effect of Na-selenite on these inflammatory parameters are under further investigation.

The present study shows that a prophylactic selenium treatment reduces the acute DBTC- induced pancreatitis and the toxicity of DBTC on biliopancreatic duct and liver. The data presented here point to a decrease of lipid peroxidation, characterized by lowered MDA levels in scope of DBTC-induced pancreatitis, following prophylactic application of selenium.

These results are confirmed by clinical studies, indicating a better outcome of patients with acute pancreatitis after a treatment with selenium (Kuklinski et al., 1991[13], 1995[14]; Wollschläger et al., 1999[47]). It is possible that a prophylactic substitution with Na- selenite has a strong beneficial effect on the clinical outcome in patients with ERCP. However, there are also conflicting results. As such, other clinical investigations described no effects of selenium in the therapy of an acute pancreatitis (Birk et al., 1995[3]; Lindner et al., 2004[16]; Steiner, 2011[41][42]). In account of these studies more experimental and clinical examinations are needed.

## Acknowledgements

The authors wish to thank Mrs. Rita Kleinfeldt and Miss Helga Rhode for their technical assistance.

## Figures and Tables

**Table 1:**
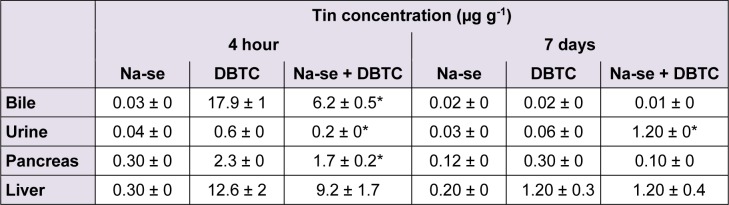
Concentrations of tin (µg g^-1^) in bile, urine, pancreas and liver 4 hours and 7 days after daily administration of Na-selenite (Na-se; 5 mg kg^-1 ^b.w. p.o.), after single dose of DBTC (6 mg kg^-1 ^b.w. i.v.) and after daily administration of Na-selenite (5 mg kg^-1 ^b.w. p.o.) + DBTC (6 mg kg^-1 ^b.w. i.v.). Data indicate mean values ± SD of n = 6 rats in each group, *P < 0.05 for comparison of DBTC vs. corresponding Na-selenite + DBTC group.

**Table 2:**
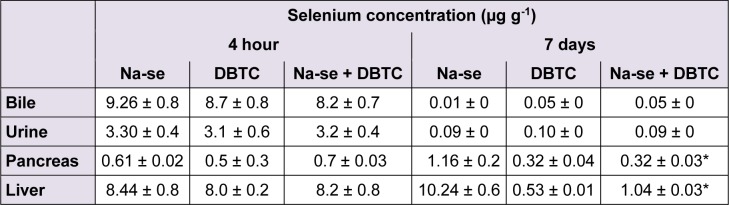
Concentration of selenium (µg g^-1^) in bile, urine, pancreas and liver 4 hours and 7 days after daily administration of Na-selenite (5 mg kg^-1 ^b.w. p.o.), after single dose of DBTC (6 mg kg^-1 ^b.w. i.v.) and after daily administration of Na-selenite (5 mg kg^-1 ^b.w. p.o.) + DBTC (6 mg kg^-1 ^b.w. i.v.). Data indicate mean values ± SD of n = 6 rats in each group, *P < 0.05 for comparison of DBTC vs. corresponding Na-selenite + DBTC group.
